# Involvement of TNF-α Converting Enzyme in the Development of Psoriasis-Like Lesions in a Mouse Model

**DOI:** 10.1371/journal.pone.0112408

**Published:** 2014-11-10

**Authors:** Kenji Sato, Mikiro Takaishi, Shota Tokuoka, Shigetoshi Sano

**Affiliations:** 1 Department of Dermatology, Kochi Medical School, Kochi University, Nankoku, Japan; 2 Pharmacology Department, Drug Research Center, Kaken Pharmaceutical Co., Ltd., Kyoto, Japan; Keio University School of Medicine, Japan

## Abstract

TNF-α plays a crucial role in psoriasis; therefore, TNF inhibition has become a gold standard for the treatment of psoriasis. TNF-α is processed from a membrane-bound form by TNF-α converting enzyme (TACE) to soluble form, which exerts a number of biological activities. EGF receptor (EGFR) ligands, including heparin-binding EGF-like growth factor (HB-EGF), amphiregulin and transforming growth factor (TGF)-α are also TACE substrates and are psoriasis-associated growth factors. Vascular endothelial growth factor (VEGF), one of the downstream molecules of EGFR and TNF signaling, plays a key role in angiogenesis for developing psoriasis. In the present study, to assess the possible role of TACE in the pathogenesis of psoriasis, we investigated the involvement of TACE in TPA-induced psoriasis-like lesions in K5.Stat3C mice, which represent a mouse model of psoriasis. In this mouse model, TNF-α, amphiregulin, HB-EGF and TGF-α were significantly up-regulated in the skin lesions, similar to human psoriasis. Treatment of K5.Stat3C mice with TNF-α or EGFR inhibitors attenuated the skin lesions, suggesting the roles of TACE substrates in psoriasis. Furthermore, the skin lesions of K5.Stat3C mice showed down-regulation of tissue inhibitor of metalloproteinase-3, an endogenous inhibitor of TACE, and an increase in soluble TNF-α. A TACE inhibitor abrogated EGFR ligand-dependent keratinocyte proliferation and VEGF production in vitro, suggesting that TACE was involved in both epidermal hyperplasia and angiogenesis during psoriasis development. These results strongly suggest that TACE contributes to the development of psoriatic lesions through releasing two kinds of psoriasis mediators, TNF-α and EGFR ligands. Therefore, TACE could be a potential therapeutic target for the treatment of psoriasis.

## Introduction

Psoriasis is one of the most common inflammatory disorders and affects >2% of the population in Western countries. It has been demonstrated that TNF-α is involved in the development of psoriasis, as evidenced by the therapeutic efficacy of TNF-α inhibitors on psoriasis [1], [2]. TNF-α has multiple functions and is one of the most important proinflammatory cytokines in psoriasis, linking innate immunity to adaptive immunity [3]. Indeed, previous studies suggested that TNF-α production from dendritic cells (DCs) is essential for activation of the pathogenic IL-23/Th17 axis in psoriasis [4].

TNF-α is produced as a membrane-bound form and is processed by TNF-α converting enzyme (TACE) to become a soluble form that exerts biological activity [5]–[7]. In addition to TNF-α, membrane-bound EGFR ligands, including amphiregulin, heparin-binding EGF (HB-EGF) and transforming growth factor (TGF)-α, are TACE substrates. More importantly, these EGFR ligands are known to contribute to the pathogenesis of psoriasis [8]–[10]. Furthermore, TACE is expressed by epidermal keratinocytes and inflammatory cells in the dermis in psoriatic lesions [11]. However, it remains unclear whether TACE is involved in the pathogenesis of psoriasis.

We previously reported that Stat3 is activated in keratinocytes in the majority of human psoriatic lesions [12]. K5.Stat3C transgenic mice, in which Stat3 is constitutively active in keratinocytes, develop psoriasis-like lesions following wounding stimuli or topical treatment with the tumor promoter 12-*O*-tetradecanoylphorbol-13-acetate (TPA), which strongly suggests that Stat3 activation is required for the development of psoriasis. The skin lesions of K5.Stat3C mice closely resemble psoriasis and provide a relevant animal model of psoriasis based on clinical, histological, immunophenotypic and biological criteria [12], [13]. For example, the skin lesions in K5.Stat3C mice show epidermal hyperplasia, infiltration of immune cells into the dermis and abscess formation in the epidermis, which represent shared pathologic features with human psoriasis [12], [14]. Furthermore, the skin lesions in K5.Stat3C mice are attenuated by administration of an anti-IL-17A antibody or anti-IL-12/23p40 antibody, similar to human psoriasis [14]. Therefore, K5.Stat3C mice provide a platform for screening potential therapeutic targets for the treatment of psoriasis.

Angiogenesis is a hallmark of psoriasis and the psoriasis-like skin lesions in K5.Stat3C mice [12]. VEGF plays a key role in angiogenesis and wound healing [15], and is a potential target for the treatment of psoriasis [16]. Upon wounding, keratinocytes produce VEGF, which is also strongly up-regulated in the epidermis of psoriatic lesions [17]. Previous studies have demonstrated that VEGF production by keratinocytes is regulated by TNF-α or HB-EGF [18], [19]. Therefore, it is likely that TACE plays a role in VEGF production from keratinocytes not only during wound healing but also in psoriasis. In this regard, TACE is a post-translational regulator for the release of multiple soluble mediators required for psoriasis.

In the present study, we investigated the expression of TACE and its related molecules in psoriasis-like skin lesions in K5.Stat3C mice, and addressed the question as to how TACE inhibition impacts the release of cytokines/growth factors and keratinocyte proliferation. The sum of our results suggests TACE inhibition as a potential strategy for the treatment of psoriasis.

## Materials and Methods

### Patients and normal controls

The study protocol was conducted in accordance with the guidelines of the World Medical Association’s Declaration of Helsinki and was approved by the Institute Ethical Review Board of the Kochi Medical School, Kochi University. Written informed consent was obtained from subjects after explaining the purpose of the study.

### Mice

All experimental procedures performed on mice were approved by the Institutional Animal Care and Use Committee of Kochi Medical School. K5.Stat3C mice were generated as previously reported [20]. Briefly, Stat3C cDNA (a gift from Dr. J. Bromberg, Memorial Sloan Kettering Cancer Center) was ligated into the pBK5 construct, followed by digestion with EcoRI. The construct was then used to generate transgenic founder mice on an FVB/N background.

### TPA-induced psoriasis-like lesions in the ears of K5.Stat3C mice

The generation of psoriasis-like lesions in the ears of K5.Stat3C mice was conducted as previously described [14], [21]. In brief, the skin lesions were generated by topical application of 0.68 nmol TPA (Wako) in 20 µl acetone to all surfaces of the left and right ears at day 0 and 2. Etanercept (Pfizer) (1 mg/mouse) was intravenously injected on day 0 and 2. The dosage of etanercept was decided as previously described [22]. AG1478 (LC laboratories) was dissolved in acetone to prepare a 0.016% solution. Following this, 20 µl AG1478 solution was topically applied to all surfaces of the left and right ears twice a day. The concentration of AG1478 was decided as previously described [23]. Ear thickness was measured every day from day 0 to 3. The mice were sacrificed and ear skins were collected for gene and protein expression analysis and histological analysis. All mouse experiments were performed with strict adherence to institutional guidelines for minimizing distress.

### Histology

Ear tissues were fixed in 20% formalin and then embedded in paraffin. Sections were obtained from the paraffin blocks and stained by hematoxylin and eosin (H&E) using standard methods. Epidermal thickness was measured at 12 spots in the interfollicular epidermis in each slide.

### Immunohistochemical staining

Formalin-fixed slides were deparaffinized with xylene and rehydrated in an alcohol gradient. Slides were autoclaved in 10 mmol/l citrate (pH 6.0) at 115°C for 5 min to retrieve antigen, then incubated for 40 min at room temperature. Endogenous peroxidase was quenched using 3% hydrogen peroxide, then non-specific antibody reaction was blocked using protein block serum free (DAKO) at room temperature for 30 min. Slides were incubated with specific primary antibodies, rabbit anti-human TACE antibody (QED Bioscience) or rabbit anti-human TNF-α (IHC-world), at 4°C overnight. Slides were washed with PBS and subjected to horseradish peroxidase-conjugated secondary antibody (DAKO) for 30 min at 37°C. The slides were washed with PBS, then detected by diaminobenzidine substrate kit (Life Technologies). All slides were counterstained with hematoxylin.

### Quantitative real-time PCR

Ear tissues were minced with scissors into small pieces on ice, and were then disrupted by sonication in RNA lysis buffer contained in an RNA isolation kit (Promega). For some experiments, primary keratinocytes were stimulated with 20 ng/ml IL-17A (R&D Systems) and 10 ng/ml TNF-α (R&D Systems) for 24 h and peritoneal macrophages were stimulated with 100 ng/ml LPS (Sigma) for 0.5, 6 and 24 h pretreated with or without 10 µmol/l TAPI-1 (Enzo Life Sciences), for 30 min prior to stimulation. Total RNAs were extracted using an RNeasy Mini kit (Qiagen) according to the manufacturer’s protocol. RNAs were reverse transcribed using M-MLV reverse transcriptase with random oligonucleotide hexamers (Life Technologies). Quantitative PCR reactions were performed using Taqman Master Mix or Power SYBR Green PCR Master Mix (Life Technologies). Amplification conditions were as follows: 50°C for 2 min, 90°C for 10 min for 1 cycle, followed by 40 cycles of 95°C for 15 sec and 60°C for 1 min. Primers used were described elsewhere [14] and those for HPRT (Mm01545399_m1), TNF-α (Mm00443258_m1), amphiregulin (Mm00437583_m1), HB-EGF (Mm00439306_m1), TGF-α (Mm00446232_m1) and TIMP-3 (Mm00441826_m1), all from Life Technologies. The quantity of each transcript was analyzed using the 7300 Fast System Software (Life Technologies) and was normalized to hypoxanthine phosphoribosyltransferase (*HPRT*), according to the ΔΔ Ct method.

### Determination of TNF-α in skin samples

For measurement of TNF-α in skin, ear biopsy samples were taken using a punch (a diameter of 8 mm, Kai Industries Co. Ltd.). Ear tissues were minced with scissors into small pieces on ice in cold PBS containing protease inhibitor cocktail, phosphatase inhibitor cocktail 2 and 3 (Sigma), and were disrupted by sonication. The homogenized tissues were centrifuged at 20,000 g for 20 min at 4°C, and supernatants were harvested. TNF-α in the supernatants was measured using ELISA for TNF-α (R&D Systems).

### Measurement of in vivo TACE activity

TACE activity was determined by the SensoLyte 520 TACE Activity Assay Fluorimetric Kit (AnaSpec), according to the manufacturer’s instructions. In brief, Ear skins were minced with scissors into small pieces in cold assay buffer containing 0.1% TritonX-100 and were homogenized. The lysates were centrifuged at 2,000 g for 15 min at 4°C, and supernatants were harvested. TACE activity from 25 µg of total protein was measured using the kit for TACE activity.

### Western blot

The protein extraction from ear skins was performed as described above. The protein concentration of each supernatant was measured with the Bio-Rad protein assay system (Bio-Rad). The supernatants were separated on 4–20% SDS/PAGE according to the method as previously described [24], under reducing conditions. Recombinant TNF-α (eBioscience) was loaded as a positive control in some experiments. Separated proteins were electrophoretically transferred to polyvinylidene difluoride membranes and blocked with 5% skim milk in TBS with 0.1% Tween 20 (TBST) for 1 h at room temperature. Membranes were incubated with specific primary antibodies, goat anti-mouse TNF-α antibody (Antigenix America) or rabbit anti-mouse TIMP-3 antibody (Millipore), at 4°C overnight. Membranes were washed with TBST and subjected to horseradish peroxidase-conjugated secondary antibodies against goat (SantaCruz) and rabbit (GE Healthcare) for 45 min at room temperature. The membranes were washed with TBST, then detection for TNF-α and TIMP-3 were performed using SuperSignal West Femto (Thermo Fisher Scientific) and ECL plus Western Blotting Detection System (GE Healthcare), respectively. The membranes were stripped by Restore Western blot stripping buffer (Thermo Fisher Scientific) and reprobed with mouse anti-mouse fiactin (Sigma) for 1 h at room temperature. The membranes were washed with TBST, then subjected to corresponding horseradish peroxidase-conjugated secondary antibodies (GE Healthcare). The membranes were washed with TBST, then detected with ECL plus Western Blotting Detection System.

### Preparation of bone marrow derived dendritic cells (BMDCs), peritoneal macrophages and keratinocytes

BMDCs were generated as previously described [25]. Briefly, bone marrow cells taken from wild-type FVB/N mice were suspended in RPMI 1640 (Life Technologies) containing 10 ng/ml GM-CSF (Peprotech), antibiotic-antimycotic (Life Technologies) and 10% FBS (Nichirei Bioscience). BMDCs were harvested 9 d later and plated at 5×10^4^ cells/well in 96-well plates. Peritoneal macrophages were isolated as previously described [26] with modifications. Briefly, wild-type FVB/N mice were injected i.p. with 500 µL 4% sterile thioglycolate (Becton Dickinson) solution in PBS, and cells were harvested by peritoneal lavage 4 d later. Cells were plated at 5×10^4^ cells/well in 96-well plates. For gene expression analysis, cells were plated at 2.5×10^5^ cells/well in 24-well plates. Primary keratinocytes were isolated from the skin of K5.Stat3C newborn mice as described [12]. In brief, epidermis was isolated by overnight digestion of dorsal skin from K5.Stat3C newborn mice with dispase (Becton Dickinson) at 4°C. The epidermis was incubated with 0.25% trypsin (Life Technologies) for 5 min at 37°C. After stopping the trypsin reaction with FBS, the keratinocyte suspension was passed through a 70 µm cell strainer. Freshly isolated primary keratinocytes were plated at 2.5×10^5^ cells/well in 24-well plates for the detection TNF-ted wiamphiregulin or 1.5×10^5^ cells/well in 48-well plates for the detection VEGF in DMEM-Glutamax (Life Technologies) containing antibiotic-antimycotic and 10% FBS. Three hours later, the medium and floating cells were removed, and attached cells were refed with Epilife (Life Technologies) supplemented with Hu-Media KG (Kurabo). Cells were cultured until they were subconfluent, then used for the detection of TNF-α, amphiregulin and VEGF. For the proliferation assay, freshly isolated primary keratinocytes were plated at 6×10^4^ cells/well in 48-well plates. A medium change was performed as described above, then cells were cultured for 2 days.

### Production of TNF-α, amphiregulin and VEGF in vitro

BMDCs and peritoneal macrophages from wild-type FVB/N mice were stimulated with 100 ng/ml LPS. Primary keratinocytes isolated from K5.Stat3C newborn mice were stimulated with 100 nmol/l TPA for 24 h. Cells were pretreated with or without TAPI-1 for 30 min prior to stimulation. The concentrations of cytokines and growth factors in the culture medium were examined by ELISA for TNF-α (eBioscience), amphiregulin (R&D Systems) and VEGF (R&D Systems).

### Keratinocyte proliferation assay and Cytotoxic assay

Primary keratinocytes were treated with or without 100 µmol/l TAPI-1 or 100 nmol/l AG1478. HB-EGF (BioVision) or amphiregulin (R&D Systems) (all at 100 ng/ml) were added at 30 min after TAPI-1 or AG1478 treatment, then cultured for 3 days. Keratinocyte proliferation was assessed using 3-(4,5-dimethylthiazol-2-yl)-5-(3-carboxymethoxyphenyl)-2-(4-sulfophenyl)-2H-tetrazolium, inner salt (MTS) assay (Promega) on day 0 to 3, or bromodeoxyuridine (BrdU) ELISA (Roche) on day 2. Toxicity effects and adverse reactions to chemical compounds were assessed by measuring lactate dehydrogenase (LDH) release in the culture medium (Promega), and Triton X-100 (Wako) was used as a positive control.

### Statistical analysis

Statistical differences were evaluated by Student’s *t*-test, Tukey-Kramer’s test or Duunett’s test. *p* values less than 0.05 were considered significant.

## Results

### Expression of TACE and TNF-α in human psoriatic skin and in psoriasis-like skin lesions of K5.Stat3C mice

**Figure 1:**
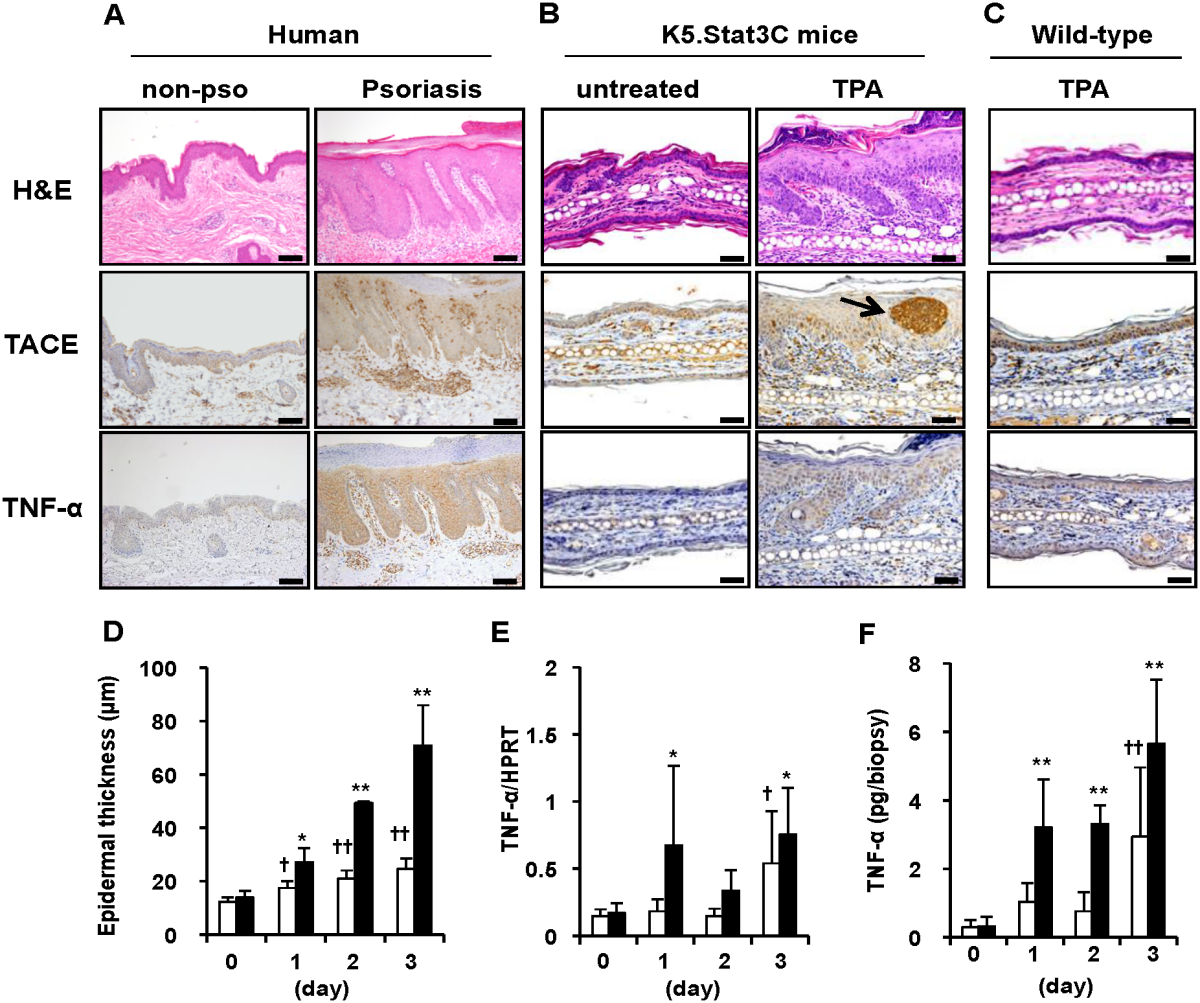
Expression of TACE and TNF-α in the development of psoriasis-like skin lesions in K5.Stat3C mice. (A–C), Representative histology and immunohistochemistry of human skins (A), ear skins in K5.Stat3C mice (B), and wild-type mice (C). non-pso, non-psoriasis control; TPA, TPA-treated ear skins sampled at day 3; Hematoxylin and eosin staining (H&E, top panels), Immunohistochemical staining for TACE (middle panels) and TNF-α (bottom panels). Arrow, intraepidermal pustule of neutrophils. Bars = 100 µm (human), 50 µm (mouse). (D–F), epidermal thickness (D), TNF-α mRNA expression (E) and TNF-α protein levels (F) in the TPA-treated ear skins of K5.Stat3C mice (black bars) and wild-type mice (white bars). Data represent means ± SD of 3 to 8 mice. *p<0.05, **p<0.01, versus K5.Stat3C mice at day 0, ^†^ *p*<0.05, ^††^ *p*<0.01, versus wild-type mice at day 0, by Dunnett’s test.

As previously described, TACE was expressed in all layers of the epidermis, blood vessels and appendages in human psoriatic lesions as well as in uninvolved human skin (Fig. 1A, middle panels) [11], [27]. However, dermal inflammatory cells in psoriatic lesions strongly expressed TACE (Fig. 1A, right middle panel) [11], [27]. TNF-α was expressed in the epidermis and inflammatory cell infiltrates of psoriatic lesions (Fig. 1A, right bottom panel), whereas uninvolved skin did not express TNF-α (Fig. 1A, left bottom panel) as previously reported [28]. Topical treatment with TPA resulted in the emergence of psoriasis-like lesions in K5.Stat3C mice, but not in wild-type mice (Fig. 1B–D) [14]. The histopathology of skin lesions in K5.Stat3C included epidermal hyperplasia, a number of inflammatory cell infiltrates in the dermis, and abscess formation in the epidermis, all of which resemble psoriasis (Fig. 1A–B, top panels). Similar to human psoriasis, TPA-induced psoriasis-like lesions in K5.Stat3C mice were positive for TACE in the epidermis, inflammatory cells, blood vessels and appendages (Fig. 1A–B, middle panels). It is noteworthy that the neutrophilic abscess in the epidermis showed strong TACE expression (Fig. 1B, arrow). Furthermore, as found in human psoriasis [28] (Fig. 1A, bottom panels), TNF-α was overexpressed in psoriasis-like skin lesions of K5.Stat3C mice, although virtually no TNF-α was found in untreated control skin (Fig. 1B, bottom panels). However, TPA treatment induced TNF-α expression in the epidermis of wild-type mice as well (Fig. 1C, bottom). This was supported by the results of RT-PCR (Fig. 1E) and ELISA (Fig. 1F) showing that TPA-treated skins of wild-type mice showed increased TNF-α expression as well as K5.Stat3C mice on day 3. Taken together, elevated levels and distribution of TACE and TNF-α in skin lesions of K5.Stat3C mice are similar to those in human psoriasis.

### TNF-α is involved in the development of psoriasis-like skin lesions in K5.Stat3C mice

**Figure 2:**
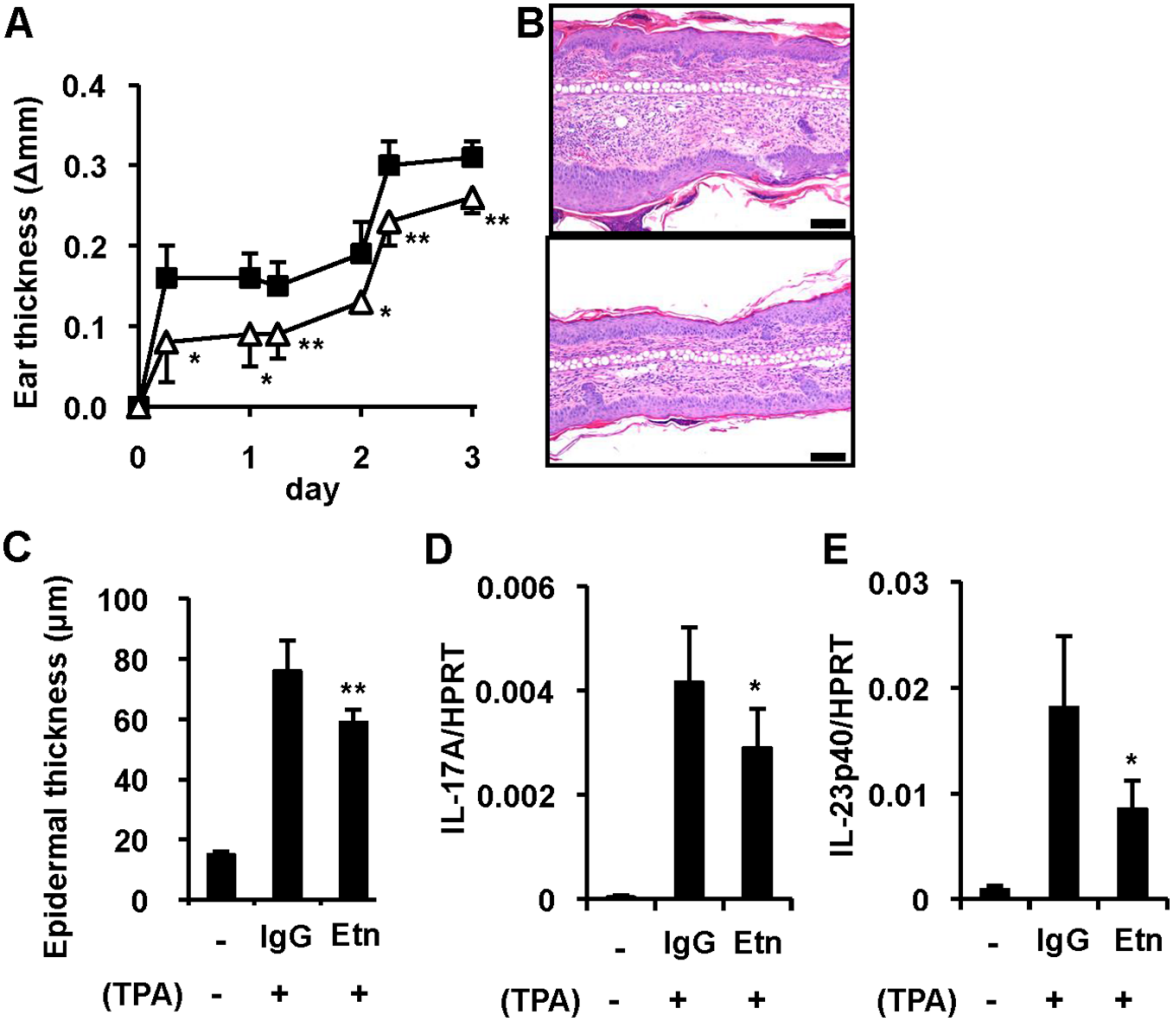
Involvement of TNF-α in the development of psoriasis-like skin lesions in K5.Stat3C mice. (A), Ear thickness (Δmm) ± SD following topical TPA in mice treated with control IgG (n = 6, squares) and etanercept (n = 6, triangles). *p<0.05, **p<0.01, versus control IgG, by Student’s *t*-test. (B), Representative histology of ear skins from K5.Stat3C mice treated with control IgG (top) and etanercept (bottom). H&E staining. Bar = 100 µm. (C–E), Inhibitory effect of etanercept (Etn) on TPA-induced epidermal hyperplasia (C), gene expression of IL-17A (D), and IL-12/23p40 (E) in ear skins. Data represent means ± SD of 3 to 6 mice. *p<0.05, **p<0.01, versus control IgG, by Student’s *t*-test.

To examine whether TNF-α is involved in the development of skin lesions, we intravenously injected a TNF-α inhibitor, etanercept, to K5.Stat3C mice before topical TPA application. Etanercept attenuated the TPA-induced thickening of ear skins (Fig. 2A) as well as epidermal hyperplasia (Fig. 2B–C). In addition, etanercept lowered the TPA-induced gene expression of IL-17A and IL-12/23p40 in the skin lesions (Fig. 2D–E). These results suggest that the inhibition of TNF-α signaling attenuates the psoriasis-like phenotype in K5.Stat3C mice through down-regulation of the IL-23/Th17 axis as found in patients with psoriasis treated with TNF-α inhibitors [4]. These results suggest that the inhibition of TNF-α attenuates the psoriasis-like phenotype in K5.Stat3C mice as occurs in patients with psoriasis treated with TNF-α inhibitors.

### EGFR signaling is involved in the development of skin lesions in K5.Stat3C mice

**Figure 3:**
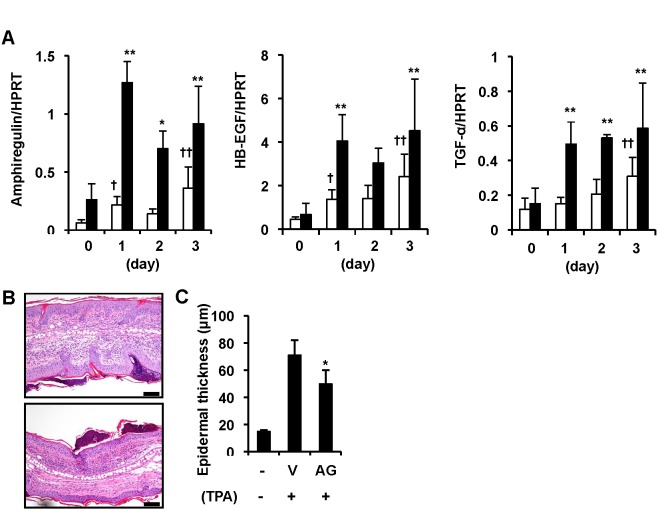
Involvement of EGFR signaling in the development of psoriasis-like skin lesions in K5.Stat3C mice. (A), Gene expression of amphiregulin, HB-EGF and TGF-α in the TPA-treated ear skins of K5.Stat3C mice (black bars) and wild-type mice (white bars). Data represent means ± SD of 3 to 8 mice. *p<0.05, **p<0.01, versus K5.Stat3C mice at day 0, ^†^ *p*<0.05, ^††^ *p*<0.01, versus wild-type mice at day 0, by Dunnett’s test. (B), Representative histology of ear skins from K5.Stat3C mice treated with vehicle alone (top) and AG1478 (bottom). Bars = 100 µm. (C), Suppression of TPA-induced epidermal hyperplasia by treatment with AG1478 (AG) at day 3. Data represent means ± SD of 3 to 5 mice, *p<0.05 versus vehicle alone (V) by Student’s *t*-test.

EGFR ligands, including amphiregulin, HB-EGF and TGF-α, all of which are TACE substrates, have been shown to be up-regulated in psoriatic lesions [29]. We analyzed the gene expression of EGFR ligands during the development of psoriasis-like skin lesions in K5.Stat3C mice. Transcriptional levels of amphiregulin, HB-EGF and TGF-α were increased at day 1, and remained elevated until day 3, whereas the increase in levels of those genes in wild-type mouse skin was much less pronounced during 3 days of topical TPA application (Fig. 3A). This result indicated that up-regulation of EGFR ligands was associated with Stat3 activation in keratinocytes [13]. To assess whether EGFR signaling is involved in TPA-induced psoriasis-like skin lesions, we topically treated K5.Stat3C mice with AG1478, an EGFR inhibitor. AG1478 attenuated the epidermal thickness (Fig. 3B–C), which suggests that the EGFR signaling by the aforementioned EGFR ligands contributed, at least in part, to the generation of lesions in K5.Stat3C mice.

### Down-regulation of TIMP-3, an endogenous TACE inhibitor, and increased TACE enzymatic activity in psoriasis-like skin lesions of K5.Stat3C mice

**Figure 4:**
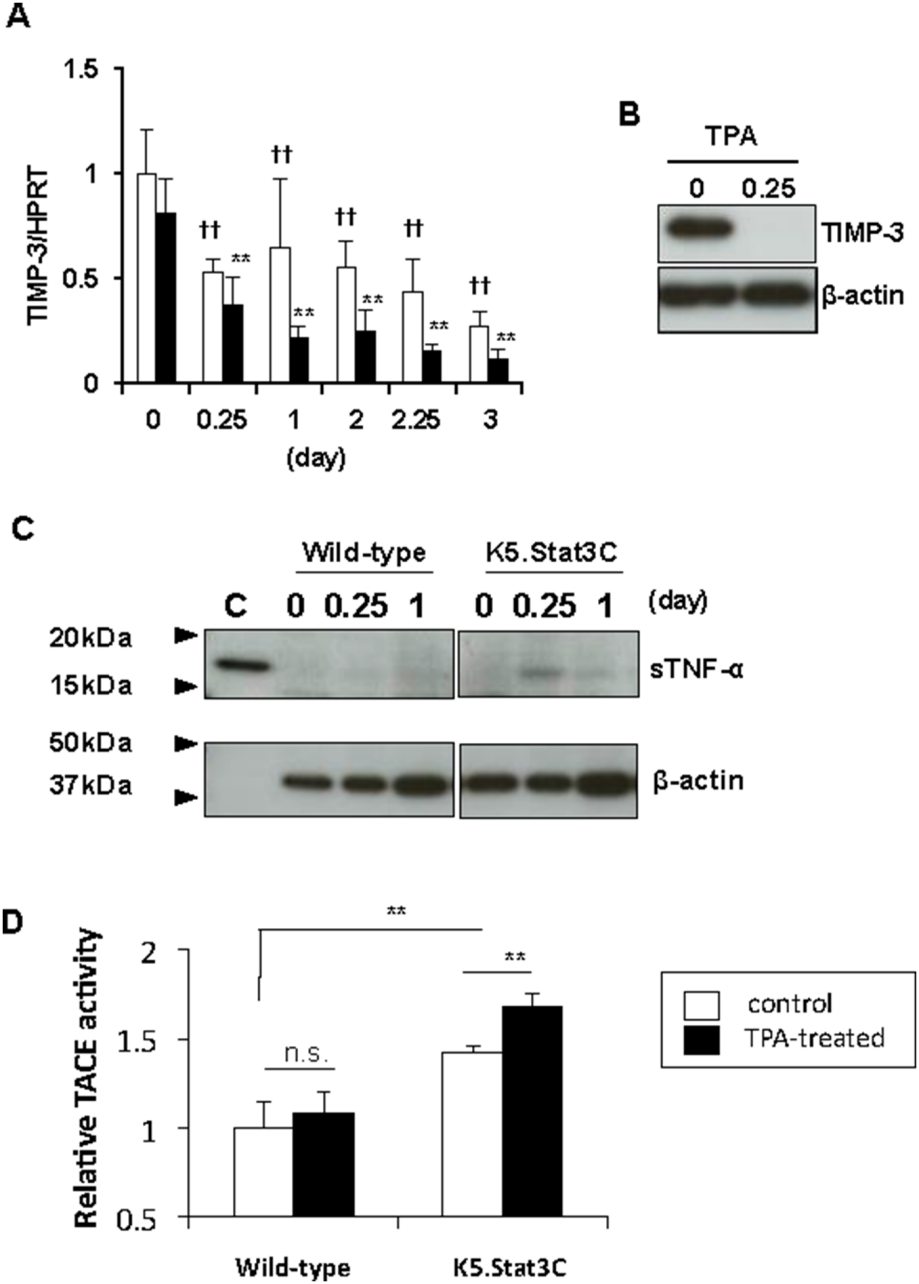
Down-regulation of TIMP-3, an endogenous TACE inhibitor, and TACE enzymatic activity in the development of psoriasis-like skin lesions. (A), Gene expression of TIMP-3 in the TPA-treated ear skins of K5.Stat3C mice (black bars) and wild-type mice (white bars). Data represent means ± SD of 3 to 8 mice. **p<0.01, versus K5.Stat3C mice at day 0, ^††^ *p*<0.01, versus wild-type mice at day 0, by Dunnett’s test. (B), Western blot analysis of TIMP-3 in ear skins of K5.Stat3C mice collected before TPA application (0) or 6 h (0.25) after TPA application. (C), Western blot analysis of soluble TNF-α (sTNF-α), which is around 17 kDa of molecular size, in the lysates from ear skins of K5.Stat3C mice compared with wild-type mice. The ear skins were collected before TPA application (0) or at 6 h (0.25) and 1 day (1) after TPA application. C, Control of soluble TNF-α. (D), Enzymatic activity of TACE in ear skins of K5.Stat3C mice versus wild-type mice; untreated control (white bars) and TPA-treated at 6h of the second TPA application (black bars). Enzymatic activity in skins was indicated as ratio to that in untreated wild-type skins. Data represent means ± SD of 4 to 5 mice, **p<0.01; n.s., not significant by Tukey-Kramer’s test.

Tissue inhibitor of metalloproteinase-3 (TIMP-3) has been reported as the negative regulator of the enzymatic activity of TACE [30]–[32]. Gene expression of TIMP-3 in the ear skin of K5.Stat3C mice was decreased in a time-dependent manner during the generation of TPA-induced lesions (Fig. 4A, black bars), while this decline was much less pronounced in wild-type mice (Fig. 4A, white bars). Strikingly, Western blot analysis revealed that TIMP-3 protein levels in the ear skin of K5.Stat3C mice were almost completely abrogated as early as 6 h of TPA application (Fig. 4B), and in turn, soluble TNF-α emerged around 17 kDa of molecular weight (Fig. 4C), through TACE-mediated processing of its membrane-bound form. In contrast, soluble TNF-α was hardly detected from the lysates of wild-type mice at this early time point (Fig. 4C). However, the gene expression of TACE was not different between wild-type mice and K5.Stat3C mice, or not increased by TPA treament (data not shown), suggesting that the post-transcriptional function of TACE might be enhanced likely by down-regulation of TIMP-3. Indeed, the skin of K5.Stat3C mice at baseline showed higher enzymatic activity of TACE than wild-type mice, and it was further increased following TPA treatment (Fig. 4D). Taken collectively, these results implicated that TACE was favorably activated in the skin of K5.Stat3C mice, at least in part, due to down-modulation of TIMP-3, leading to lesion development.

### TACE releases TNF-α from various cells

**Figure 5:**
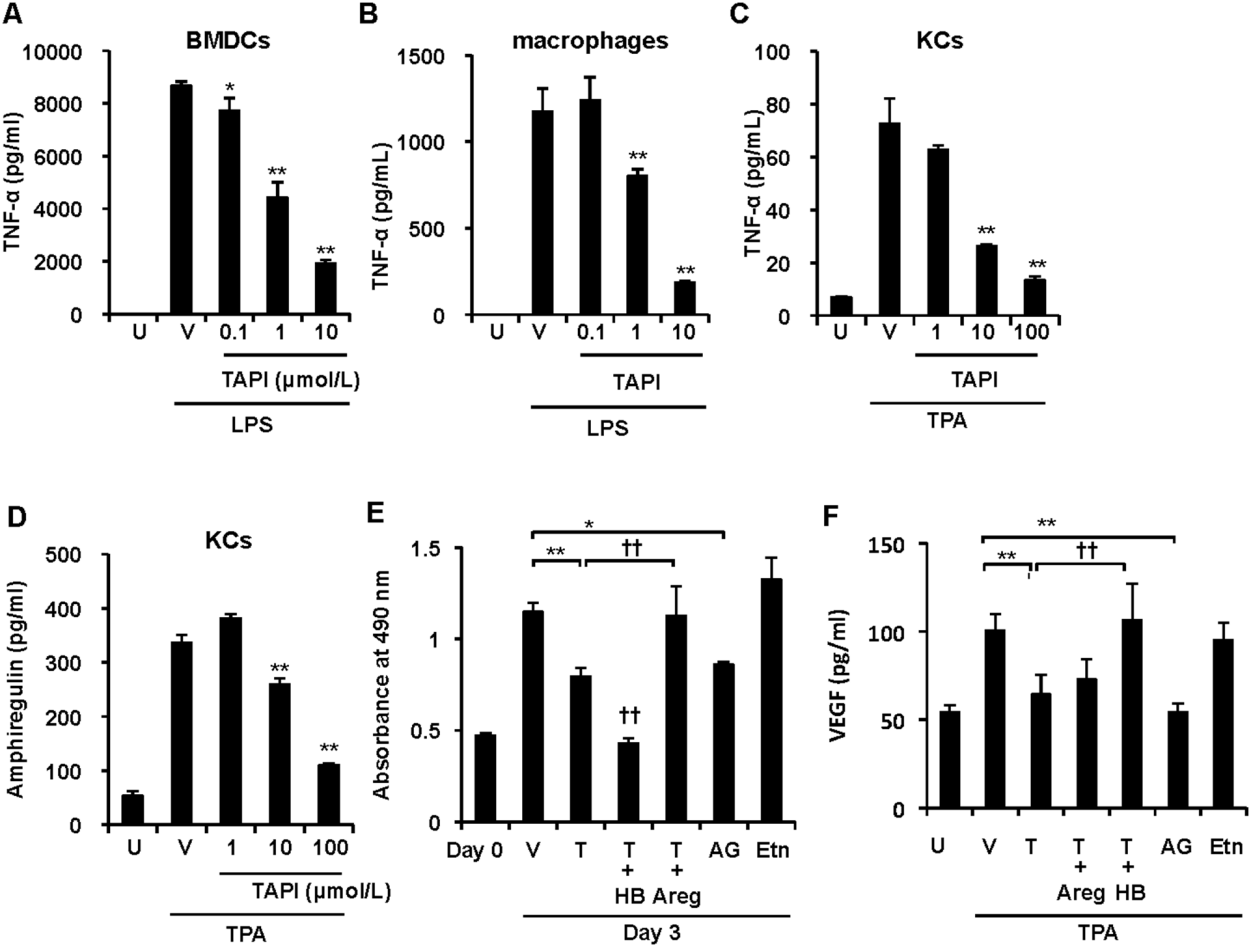
Contribution of TACE to the production of soluble TNF-α and EGFR ligands from murine cells. (A–C), TNF-α proteins in the 24 h culture supernatants of cells pretreated with or without TAPI-1 (TAPI) at the indicated concentrations. LPS-stimulated bone marrow derived dendritic cells (BMDCs) from wild-type FVB/N mice (A), LPS-stimulated thioglycolate-elicited peritoneal macrophages from wild-type FVB/N mice (B), and TPA-stimulated primary keratinocytes from K5.Stat3C newborn mice (C). Cells were pretreated with or without TAPI-1 (TAPI) for 30 min prior to stimulation. (D), Production of amphiregulin from TPA-treated primary keratinocytes of K5.Stat3C newborn mice for 24 h. Cells were pretreated with or without TAPI-1 (TAPI) at the indicated concentrations for 30 min prior to stimulation. U, unstimulated control. V, vehicle alone. Data represent means ± SD of triplicate wells. *p<0.05, **p<0.01, versus vehicle alone by Dunnett’s test. (E), Keratinocyte proliferation evaluated by MTS assay with absorbance at 490 nm. Cells were untreated or treated with TAPI-1 (100 µmol/l) (T) and cultured in the absence or presence of HB-EGF (HB), amphiregulin (Areg), AG1478 (AG) or etanercept (Etn) for 3 days. (F), VEGF production from primary keratinocytes. Cells were stimulated with TPA for 24 h treated with or without reagents as described. Data represent means ± SD from triplicate wells. *p<0.05, **p<0.01 versus vehicle alone, ^††^ *p*<0.01 versus TAPI-1 alone by Tukey-Kramer’s test.

TNF-α is produced by various cells in psoriasis, including keratinocytes, dendritic cells, macrophages and lymphocytes [33], [34]. To assess whether TACE plays a role in releasing TNF-α from these cells, we added a relatively selective inhibitor of TACE, TAPI-1 [35], to an in vitro culture of bone marrow-derived dendritic cells (BMDCs), peritoneal macrophages and primary keratinocytes. TAPI-1 significantly and dose-dependently suppressed the LPS- or TPA-induced release of TNF-α from BMDCs and macrophages from wild-type FVB/N mice and keratinocytes of K5.Stat3C newborn mice, respectively (Fig. 5A–C). The release of TNF-α from T lymphocytes was also abrogated by TAPI-1 (data not shown). It should be noted that TNF-α mRNA levels in macrophages were increased by LPS stimulation but not affected by TAPI-1 treatment (Fig. S1), verifying that TAPI-1 inhibited the release of TNF-α post-transcriptionally. Collectively, these results clearly indicate that the release of soluble TNF-α from a variety of cells is dependent on TACE activity.

### TACE induces the release of EGFR ligands from keratinocytes

EGFR ligands are produced by keratinocytes, and thereby an auto-stimulation loop is formed to generate psoriatic changes in an autocrine and paracrine fashion [10], [36]. To assess whether TACE activity is required for the release of EGFR ligands as well as TNF-α, we evaluated the effects of TAPI-1 using primary cultured keratinocytes isolated from K5.Stat3C mice. Like TNF-α, amphiregulin was released by TACE from keratinocytes in response to TPA, since it was significantly suppressed by TAPI-1 in a dose-dependent manner (Fig. 5D). EGFR stimulation leads to keratinocyte proliferation, and contributes to epidermal hyperplasia in psoriasis [29], [37] and the skin phenotypes of K5.Stat3C mice as well (Fig. 3). We next examined whether TACE is directly required for keratinocyte proliferation in response to the endogenous EGFR ligands. In vitro keratinocyte proliferation was suppressed by TAPI-1 and AG1478 (Fig. 5E). The inhibitory effects of both reagents on cell proliferation were confirmed using a BrdU incorporation assay (Fig. S2A). TAPI-1 or AG1478 did not raise lactate dehydrogenase levels in the culture supernatants compared with the vehicle control, thus ruling out cytotoxicity as a possible cause of impaired keratinocyte proliferation (Fig. S2B). The suppression of keratinocyte proliferation by TAPI-1 was reversed by the addition of amphiregulin, suggesting that TACE is involved in keratinocyte proliferation via EGFR stimulation with soluble amphiregulin (Fig. 5E). Although the underlying mechanism is not known, HB-EGF seemed to augment the TAPI-1-induced suppression of keratinocyte proliferation. VEGF is an angiogenetic factor, and is involved in the pathogenesis of psoriasis [17], [38], [39]. Psoriatic lesions in K5.Stat3C mice showed increased levels of VEGF [12] (Fig. S3). Furthermore, TAPI-1 and AG1478 inhibited the TPA-induced VEGF production from K5.Stat3C keratinocytes to a similar extent (Fig. 5F). Suppression of VEGF production by TAPI-1 was reversed by the addition of HB-EGF, but not by amphiregulin (Fig. 5F). These results demonstrate that TACE-dependent HB-EGF stimulation leads to VEGF production by K5.Stat3C keratinocytes. Taken together, TACE is required for the release of soluble EGFR ligands from keratinocytes, thereby promoting keratinocyte proliferation and VEGF production, which are required for the development of psoriasis.

## Discussion

The auto-stimulation loop of cytokine/growth factors within the epidermis and its bidirectional interaction with immunocytes play critical roles in the development of psoriasis. One of the proinflammatory cytokines, TNF-α, and growth factors of the EGFR ligand family represent essential mediators for the pathogenesis of psoriasis. TNF-α and EGFR ligands are overexpressed in plaque psoriasis compared with uninvolved skin or healthy control skin [28], [29]. Our current study highlights the role of TACE, by which both TNF-α and growth factors for EGFR are shed to form soluble ligands. Here we took advantage of K5.Stat3C mice, which develop skin lesions that closely resemble human psoriasis with respect to clinical features, histopathology, immunological abnormalities and sensitivities to biologics [12], [14], [40].

The role for TNF-α in psoriasis has been well documented, and is supported by a number of studies showing successful therapeutic effects of its inhibitors [1], [2]. It was suggested that TNF-α stimulation of DCs results in IL-23 production, which is required for Th17 cell activation [4], [41]. Indeed, a comparison of gene expression in psoriasis patients between responders to etanercept, a TNF-α inhibitor, and non-responders revealed that the responsiveness is dependent on inactivation of the Th17 immune response [42]. We showed that TNF-α stimulation enhances the production of IL-12/IL-23p40, one of the IL-23 subunits, from murine bone marrow-derived DCs (BMDCs) (data not shown). TNF-α inhibition also abrogates Th17 differentiation in vitro [43]. Furthermore, TNF-α synergistically augments IL-17A stimulation of gene expression by keratinocytes, including IL-24 (a member of IL-20 subfamily cytokines), IL-17C, TNF-α, HB-EGF, TGF-α, S100A7, S100A8, S100A9, β-defensin-3 and β-defensin-14 (Fig. S4). Thus, TNF-α stimulation impacts a variety of cells that release mediators required for the pathogenesis of psoriasis. In the experimental setting of the present study, however, treatment with etenercept (Etn) resulted in significant but insufficient attenuation of psoriatic phenotype of K5.Stat3C mice. The incomplete in vivo effect of Etn might be due to insufficient local concentrations of Etn in the skin lesions or lymphoid organs, where TNF-α was required for immune cell activation to drive psoriatic change.

EGFR signaling participates in psoriasis through its promotion of keratinocyte proliferation [44], [45], and production of growth factors such as VEGF [18], [19]. Indeed, EGFR inhibitors improve lesions in patients with psoriasis [46], [47], as also shown in skin lesions of K5.Stat3C mice in the present study. Previous studies demonstrated that topical TPA application led to EGFR activation followed by Stat3 activation in keratinocytes [48]. Therefore, it was suggested that EGFR signaling was involved in the amplification of Stat3 signaling and contributed to the development of the skin lesions in this murine model. Similar to previous studies showing that amphiregulin promotes human keratinocyte proliferation [36], [37], we showed here that the TACE inhibitor-induced attenuation of murine keratinocyte proliferation was reversed by the inclusion of amphiregulin but not by HB-EGF. Specifically, HB-EGF-mediated EGFR triggering led to VEGF production by HaCat cells [19]. VEGF plays a critical role in angiogenesis of psoriatic lesions [15], which are attenuated by treatment with an anti-VEGF antibody [39]. Likewise, we confirmed the role of HB-EGF-mediating EGFR signaling in inducing VEGF production by keratinocytes from K5.Stat3C mice, since TACE inhibition reduced the production of VEGF, which was reversed by the addition of recombinant HB-EGF, but not by amphiregulin. Thus, EGFR-mediated biological outcomes were determined by distinct ligands, all of which were sensitive to TACE inhibition. Therefore, results in the present study is, at least in part, reproduced previous studies demonstrating that TACE mediated context-dependent function of amphiregulin and HB-EGF; cell proliferation and migration possibly through VEGF signaling, respectively [37], [49].

Psoriatic lesions both in humans and in K5.Stat3C mice demonstrated a similar distribution pattern of TACE and its substrate TNF-α. Interestingly, an endogenous TACE inhibitor, TIMP-3 was found to be decreased in psoriatic lesions compared with uninvolved skin and healthy control skin [50]. It was demonstrated that mice lacking Jun-B/c-Jun in the epidermis showed a down-regulation of TIMP-3, resulting in the generation of a psoriasis-like phenotype through massive TNF-α shedding by TACE activation [32]. Likewise, TIMP-3 expression in the skin of K5.Stat3C mice was attenuated as the psoriasis-like lesions developed by TPA application. TACE enzymatic activity was elevated in the skin of K5.Stat3C mice at baseline, compared with wild-type mice, and it was further increased by TPA treatment. Since TACE mRNA levels were not elevated in K5.Stat3C mice, this finding suggested that Stat3 signaling contributed to enhanced TACE activity via post-transcriptional regulation. Most recently, it has been reported that microRNA-21 (miR-21) targeted *TIMP-3* and was functionally involved in the pathogenesis of psoriasis [51]. Most interestingly, miR-21 expression was up-regulated by the Stat3 signaling [52], supporting our data using K5.Stat3C keratinocytes, which showed acute decline of *TIMP-3* upon TPA treatment, thereby TACE activity might be elevated, followed by release of the TACE substrates. In other words, Stat3 is not only a transcriptional but post-transcriptional regulator for TACE ligands.

The results of the present study strongly suggest that TACE is involved in the pathogenesis of psoriasis through the release of TNF-α and EGFR ligands. Therefore, TACE inhibitors in clinical use may be relevant to both TNF-α and EGFR inhibitors. Our preliminary data showed that topical treatment with TAPI-1 mildly but significantly attenuated skin lesion development in K5.Stat3C mice. We are now under seeking for more potent and specific TACE inhibitors than TAPI-1, so that they should be relevant to clinical use for treatment of psoriasis.

## Supporting Information

Figure S1 **Effect of TAPI-1 treatment on TNF-α gene expression in LPS-stimulated peritoneal macrophages.** Cells were pretreated with or without 10 µM TAPI-1 (TAPI) for 30 min prior to 100 ng/ml LPS stimulation and harvested at 0.5, 6 and 24 h. Data represent means ± SD of triplicate wells.
(TIFF)

Figure S2 **Inhibition of keratinocyte proliferation by TAPI-1.** (A) BrdU incorporation of primary keratinocytes from K5.Stat3C mice. (B) LDH assay using keratinocyte culture supernatants. Cells were treated with or without 100 µmol/l TAPI-1 (TAPI), 100 nmol/l AG1478 (AG) or 1% TritonX-100 (Triton) and cultured for 2 days. BrdU incorporation was quantified by ELISA. Data represent means ± SD of triplicate wells. **p<0.01, versus vehicle alone (V), by Dunnett’s test. n.s., not significant.
(TIFF)

Figure S3 **VEGF production in the skin lesions of K5.Stat3C mice.** VEGF production was determined by ELISA using untreated or TPA treated ear skins. TPA treated skins were sampled at day 3. Data represent means ± SD of 4 to 5 mice. **p<0.01, versus the untreated group, by Student’s *t*-test.
(TIFF)

Figure S4 **Expression of psoriasis-related genes in primary murine keratinocytes by synergistic stimulation with TNF-α and IL-17A.** Primary keratinocytes were stimulated with 20 ng/ml IL-17A and 10 ng/ml TNF-α for 24 h. Data represent means ± SD of triplicate wells. **p<0.01, by Tukey-Kramer’s test.
(TIFF)
