# Correction: Involvement of TNF-α Converting Enzyme in the Development of Psoriasis-Like Lesions in a Mouse Model

**DOI:** 10.1371/journal.pone.0124989

**Published:** 2015-04-13

**Authors:** Kenji Sato, Mikiro Takaishi, Shota Tokuoka, Shigetoshi Sano

There is an error in the third to last sentence of the “Western blot” subsection of the Materials and Methods. The correct sentence is: The membranes were stripped by Restore Western blot stripping buffer (Thermo Fisher Scientific) and reprobed with mouse anti-mouse β-actin (Sigma) for 1 h at room temperature.

There is an error in the fifth to last sentence of the “Preparation of bone marrow derived dendritic cells (BMDCs), peritoneal macrophages and keratinocytes” subsection of the Materials and Methods. The correct sentence is: Freshly isolated primary keratinocytes were plated at 2.5×10^5^ cells/well in 24-well plates for the detection TNF- α amphiregulin or 1.5×10^5^ cells/well in 48-well plates for the detection VEGF in DMEM-Glutamax (Life Technologies) containing antibiotic-antimycotic and 10% FBS.
